# Osteocalcin, Osteopontin and RUNX2 Expression in Patients’ Leucocytes with Arteriosclerosis

**DOI:** 10.3390/diseases9010019

**Published:** 2021-03-12

**Authors:** Jörg Ukkat, Cuong Hoang-Vu, Bogusz Trojanowicz, Artur Rebelo

**Affiliations:** Department of Visceral, Vascular and Endocrine Surgery, Martin Luther University, 06120 Halle-Wittenberg, Germany; cuong.Hoangvu@uk-halle.de (C.H.-V.); bogusz.trojanowicz@uk-halle.de (B.T.)

**Keywords:** osteocalcin, osteopontin, RUNX2, arteriosclerosis, vascular

## Abstract

Introduction: Calcification is a highly relevant process in terms of development of cardiovascular diseases, and its prevention may be the key to prevent disease progression in patients. In this study we investigated the expression of osteocalcin (OC), osteopontin (OPN) and RUNX2 in patients’ leukocytes and their possible role as diagnostic markers for cardiovascular diseases. Materials and Methods: Leucocytes from 38 patients were collected in the Department of Surgery of Martin-Luther-University Halle, including 8 patients without arteriosclerotic disease (PAD−) and 30 patients with symptomatic arteriosclerotic disease (PAD+). Patients’ leucocytes, in vitro calcified human umbilical vein endothelial cells (HUVEC) and vascular smooth muscle cells (VSMC) were subjected to qPCR analyses with TaqMan probes, which are specific for OC, OPN and RUNX2. Additionally, the interaction between monocytes and calcified HUVEC and VSMC was investigated in adhesion assays. Results: The leucocytes obtained from patients with symptomatic arteriosclerotic disease (PAD+) demonstrated decreased mRNA level expression of Osteocalcin, while OPN and RUNX2 were significantly upregulated in comparison to asymptomatic patients. The induction of calcification in HUVEC and VSMC cells led to an increased expression of OC, OPN and RUNX2. Immunocytochemistry of calcified HUVEC and VSMC revealed stronger expression of OC, OPN and RUNX2 in calcified cells. Conclusion: To conclude, these data demonstrate that symptomatic arteriosclerotic disease has a correlation with OC, OPN and RUNX2. The biological rationale of OC, OPN and RUNX-2 remains not yet entirely understood for atherosclerotic disease, which means it needs further investigation.

## 1. Introduction

The arterial deposition of hydroxyapatite mineral is related to an increased risk of heart disease and stroke [1]. Cardiovascular disease represents an important cause of mortality worldwide [2]. Prevention is a key for fighting this disease [3,4]. Active reprogramming of vascular smooth muscle cells (VSMC) is described as having a crucial role in the calcification process [5].

The bone is not only a protective and static organ, but also an organ with endocrine functions. Osteocalcin (OC) is a bone γ-carboxyglutamic acid (Gla) protein produced by osteoblasts. In addition, the bone has been identified as a stress organ and osteocalcin as a stress hormone [6,7].

The collagen-rich matrix that composes bone tissue is produced by osteoblasts. In addition, other regulating functions of these cells are being studied. In the center of this potential hormonal role is Osteocalcin. These functions include mineralization, regulation of glucose and energy metabolism and regulation of fertility and cognition. Among these functions, osteocalcin was recently described as a potential marker of subclinical atherosclerosis [8].

Osteopontin (OPN) is also a protein involved in the mineralization and calcification processes. The role in inflammatory processes was studied in recent years. Multifunctional roles on physiological and pathophysiological processes also were studied in past years [9,10,11]. Additionally, OPN has also been associated with a promoter of arteriosclerosis [12]. Furthermore, osteopontin could be a target in treating coronary artery disease [13].

RUNX2 is a transcriptional regulator of skeletogenesis and it is expressed in mouse and human skeletal progenitors [14,15]. The role of RUNX2 was a matter of several studies in past years [16,17].

There is no clear definition of the value of OC, OPN and RUNX2 expression in symptomatic atherosclerotic disease. We investigated in this study the expression of OC, OPN and RUNX2 in vascular smooth muscle cells and leucocytes, and their possible role as diagnostic markers.

## 2. Material and Methods

*Patients and tissue preparation.* A total of 38 vessel tissues, including 8 patients without arteriosclerotic disease (PAD−) and 30 patients with symptomatic arteriosclerotic disease (PAD+) were collected in the Department of Surgery of Martin-Luther-University Halle.

This study was approved by the ethical committee of the Martin Luther University, Faculty of Medicine, and all patients gave written consent.

*Cell culture and calcification induction.* HUVEC (human umbilical vein endothelial cells) cell line was cultured in EGM Basalmedium (Lonza, Basel, Switzerland) and EGM Bullet Kit (Lonza). VSMC (vascular smooth muscle cells, Lifeline Cell Technology) were cultured in Ham’s F-12K Medium (Thermo Fisher Waltham, MA, USA) supplemented with 0.05 mg/mL Ascorbic acid (Sigma-Aldrich, St. Louis, MO, USA), 0.01 mg/mL Insulin (Sigma-Aldrich), 0.01 mg/mL Transferrin (SERVA), 10 ng/mL Sodium selenite (Sigma-Aldrich), 0.03mg/mL Endothelial Cell Growth Supplement (ECGS) (Sigma-Aldrich), 10 mM HEPES (Sigma-Aldrich), 10 mMTES (Sigma-Aldrich) and 10% FCS (Biochrom AG). Calcification medium was prepared by supplementation of the growth medium with 1.58 mM/L Calcium d-gluconate monohydrate (Sigma-Aldrich), 0.1 µM/L Dexamethason (Serva, Heidelberg, Germany), 0.5 mM/L Ascorbic acid (Sigma-Aldrich), 2.5 mM/L ß Glycerophosphate disodium salt hydrate (Sigma-Aldrich). HUVEC and VSMC were calcified for 7 and 14 days, respectively. There after Alizarin S staining: (Alizarin-Red Staining Solution) (Sigma-Aldrich) and ALP Assay: (Amplite (TM) Colorimetric Alkaline Phosphatase Assay Kit) (Biomol), both according to manufacturer’s instructions, were performed. Control cells received normal growth medium. Briefly, Alizarin S staining was performed on the cells seeded in 24-Well Plates in corresponding media. Before the staining medium was completely removed, the cells were shortly washed with double distilled water. Thereafter, the cells were fixed 10 min with 10% formalin in PBS and washed again three times with double distilled water. After this step the cells were stained 30 min with 40 mM Alizarin S, pH 4.1. Finally, the cells were washed again three times with double distilled water and photographed with light microscope (Axiovision, Zeiss, Oberkochen, Germany).

*RT-PCR.* Blood samples for mRNA analysis were drawn into Tempus™ Blood RNA Tubes (Thermo Fisher Waltham, Massachusetts, U.S.A.) prefilled with RNAse inhibitor and frozen at −20 °C. Total RNA was isolated with Tempus Spin RNA Isolation Kit (Thermo Fisher Scientific). Total RNA from cell culture experiments was isolated with Trizol (Invitrogen, Carlsbad, CA, USA) according to manufacturer’s instructions.

Then, 500 ng of total RNA was reversely transcribed with Superscript II kit (Gibco, Munich, Germany) at 42 °C for 30 min followed by enzyme inactivation at 95 °C for 5 min. The samples were stored at −20 °C until further processing.

QPCR reactions for OC were performed with Rotor-Gene System (Qiagen) and qPCRBIO Probe Mix (Nippon Genetics, Düren, Germany). Samples were amplified as double replicates by employment of TaqMan Assays specific for OC (Hs01587814_g1 BGLAP) and ACTB (Hs99999903-m1 ACTB). Thermal cycling conditions for TaqMan were as follows: hold 10 min 95 °C, 40 cycles of 10 s/95 °C and 30 s/60 °C. OPN and RUN X2 were amplified with 5× HOT FIREPol EvaGreen qPCR Mix (Solis Biodyne, Tartu, Estonia), specific primers mentioned in Table 1 and under following conditions: hold 10 min at 95 °C, followed by 40 cycles of 15 s at 95 °C, 30 s at 60 °C and 30 s at 72 °C. Normalization was performed with primers specific for ACTB, GAPDH and 18S (Table 1). For evaluation, each patient sample with amplified target gene was normalized to ACTB and/or GAPDH and divided by internal calibrator (positive control) to obtain the fold change value (RQ). For the whole study, the same internal calibrator, the same negative controls and the same normalizing markers were employed. Data evaluation was performed with DataAssist Software (3.01) (Life Technologies, Carlsbad, CA, USA).

*Immunocytochemistry.* 1 × 10^5^ cells were seeded on the microscopic slides (Thermo Fisher Scientific) and let grow in corresponding medium. Thereafter the cells were fixed in a 1:4 mixture of 3% H_2_O_2_ in ice cold 90% methanol for 20 min. After washing twice with PBS, cells were incubated overnight at 4 °C with the antibodies against Osteocalcin, Osteopontin and RUNX2 (all from Abcam), diluted 1:1000 with Dako Antibody Diluent (Dako, Glostrup, Denmark). Negative control sections were exposed to the secondary antibody only and processed as described below. After 3 × 10 min washing in PBS, cells were incubated for 30 min with a 1:1000 dilution of biotinylated secondary antibodies (Dako, Jena, Germany) followed by incubation with an avidin–biotin–peroxidase complex (Dako). After 3 × 10 min washing in PBS, specific immunostaining was visualized with diaminobenzidine chromogenic solution (Dako, 1:50).

Finally, cells were lightly counterstained with Mayer’s haematoxylin. Microscopic investigations were performed with light/fluorescence microscope (Biozero BZ-9000, Keyence, Osaka, Japan).

*Adhesion assay.* Adhesion of human THP-1 monocytes to calcified HUVEC or VSMC monolayers was investigated in 24-well plates. Briefly, HUVEC or VSMC cells were cultured in calcification for 7 days. For labeling, THP-1 cells were incubated in RPMI containing 1 µM calcein-AM at 37 °C for 60 min. The cells were washed with RPMI twice. Fluorescence-labeled THP-1 cells were resuspended in 2 ml RPMI medium and added (1 × 10^6^ cells/well) to control or calcified cells. The plates were incubated for 30 min at 37 °C. After incubation, the monolayer was gently washed three times with RPMI. Adherent monocytes were photographed using a Biozero BZ-9000 fluorescence microscope (Keyence). The number of the adherent cells was evaluated in 10 microscopic fields for each situation by employment of ImageJ software (Wayne Rasband, National Institutes of Health, Bethesda, MD, USA). All experiments were repeated at least three times.

*Statistics.* Data are presented as medians according to Tukey method. Distribution of the quantitative variables was tested using D’Agostino–Pearson omnibus, Shapiro–Wilk or Kolmogorow–Smirnow normality tests. Depending on data distribution, parametric (differences between paired values are consistent) or nonparametric (Wilcoxon matched pairs signed rank test) two-sided *t* tests were used; *p* < 0.05 was considered to represent statistically significant differences. GraphPad Prism (6.5) and SPSS (21) software were used for statistical analyses.

## 3. Results

### 3.1. Expression of Leucocytic OC Transcripts Is Significantly Reduced in Patients with Arteriosclerotic Disease

**Figure 1:**
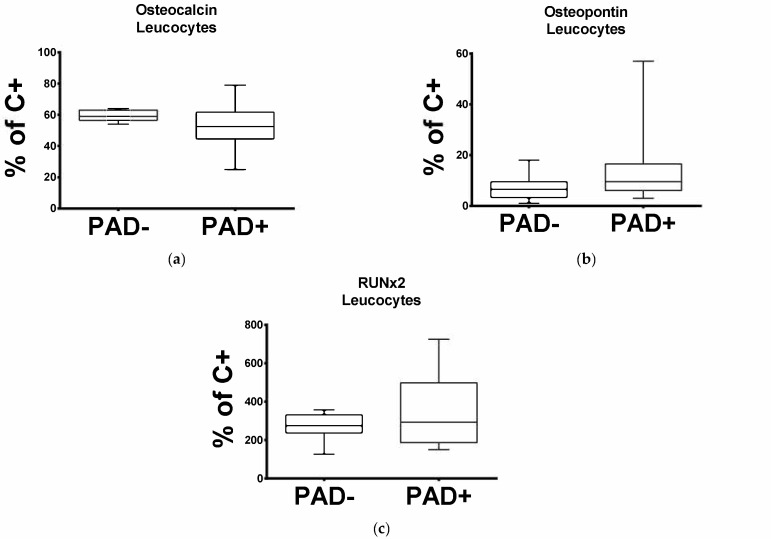
Expression of osteocalcin (OC) (**a**), osteopontin (OPN) (**b**) and RUNX2 (**c**) in leucocytes obtained from the PAD− and PAD+ groups.

In order to investigate the mRNA level pattern of OC in the cells circulating in patients with arteriosclerotic disease, we subjected the leucocytes obtained from PAD− and PAD+. to qPCR. As demonstrated in Figure 1a, OC expression is significantly reduced in patients with arteriosclerotic disease. The median percentage mRNA level for OC in PAD+ was 52.5% and for PAD− 59% (*p* = 0.0173).

### 3.2. Expression of Leukocytic OPN and RUNX2 Transcripts Is Significantly Increased in Patients with Arteriosclerotic Disease

Regarding the leucocytic OPN and RUNX2 transcripts, this was significantly elevated in PAD+ patients compared to the PAD− group (Figure 1b,c). The median percentage mRNA level for OPN in PAD+ was 9.5% and for PAD− 6.5% (*p* = 0.0241), and for RUNX2 was 292.5% and for PAD− 274.5% (*p* = 0.0449).

### 3.3. Alkaline Phosphatase Activity Was the Same in HUVEC and Increased in VSMC Cells Treated with Calcification Medium

The alkaline phosphatase activity in HUVEC cells with calcification induction was after 7 days 51 mU/mL (SD ± 0.003) and in the control cells was 51 mU/mL (SD ± 0.006). After 14 days, this was 61 mU/mL (SD ± 0.002) and in the control cells was 63 mU/mL (SD ± 0.016).

**Figure 2:**
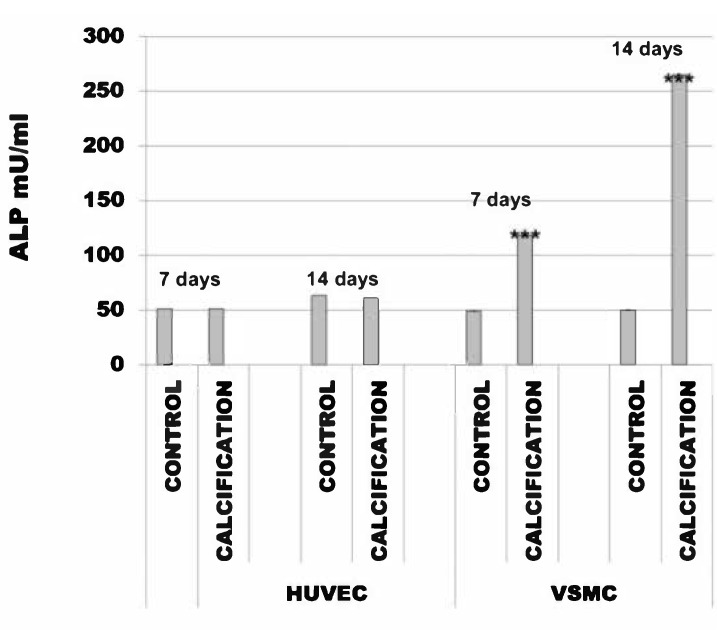
Alkaline phosphatase activity (ALP) in human umbilical vein endothelial cells (HUVEC) and vascular smooth muscle cells (VSMC) cells treated with calcification medium. Note that calcification does not alter ALP activity in HUVEC cells, *** *p* < 0.001.

The alkaline phosphatase activity in VSMC cells with calcification induction was after 7 days 118 mU/mL (SD ± 0.22) and in the control cells was 49 mU/mL (SD ± 0.03). After 14 days, this was 265 mU/mL (SD ± 0.22) and in the control cells was 50 mU/mL (SD ± 0.03) (Figure 2).

### 3.4. Induction of Calcification in HUVEC and VSMC Cells Led to Increased Expression of OC, OPN and RUNX2

The median percentage mRNA level for OC, OPC and RUNX2 in HUVEC cells with calcification induction was 92% (SD ± 9.4), 183% (SD ± 15.8) and 164% (SD ± 15.8), respectively, and in the control cells was 100% (SD ± 4.5).

**Figure 3:**
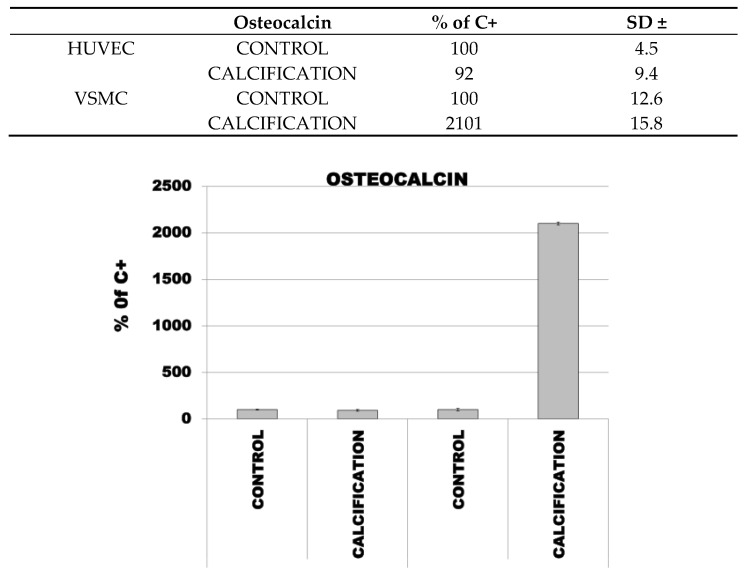
Expression of OP, OPN and RUNX2 in control and calcified HUVEC and VSMC.

The median percentage mRNA level for OC, OPC and RUNX2 in VSMC cells with calcification induction was 2101% (SD ± 15.8), 246% (SD ± 15.8) and 166% (SD ± 15.8), respectively, and in the control cells was 100 % (SD ± 12.6) (Figure 3).

### 3.5. Adhesion of THP-1 Monocytes to Calcified HUVEC or VSMC Cells Was Higher in Calcified Cells as Compared to Controls

**Figure 4:**
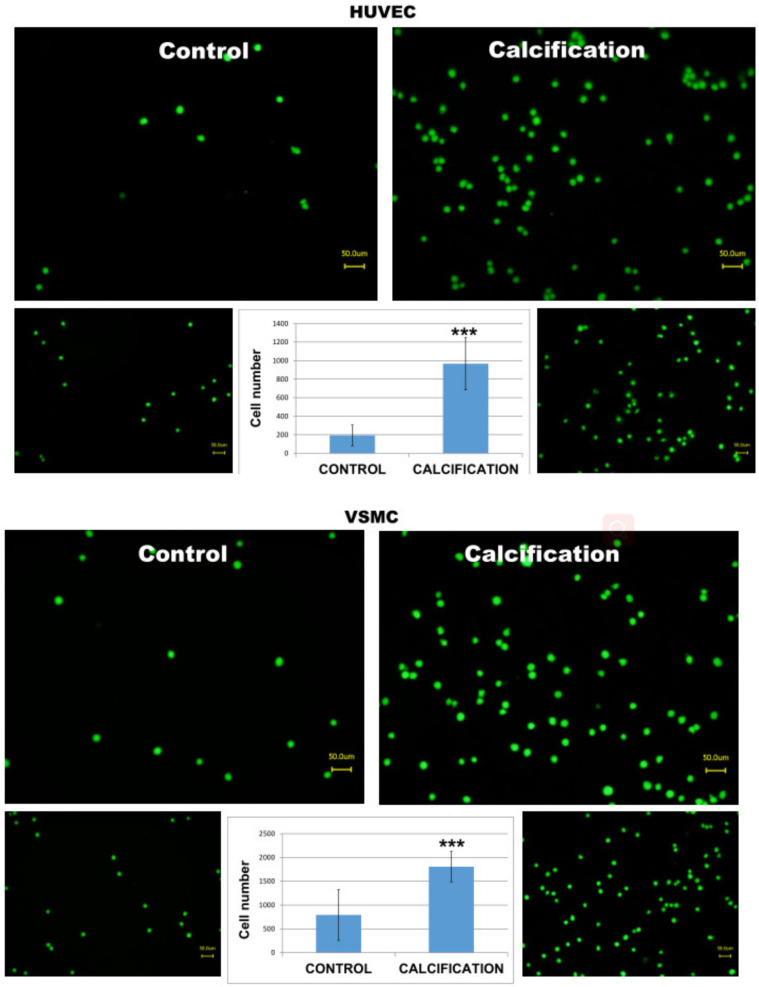
Adhesion of THP-1 monocytes to calcified HUVEC or VSMC cells. Note that a higher number of adhered monocytes is visible in calcified cells as compared to controls. *** means *p* < 0.001.

In order to test whether calcified HUVEC or VSMC cells may induce stronger adhesion and mimic increased inflammatory reaction, we incubated human THP-1 monocytes with calcified monolayers of these two cell types. (Supplementary Materials) As demonstrated in Figure 4, monocytes responded with a significantly higher number of adhered cells as compared to uncalcified control HUVEC or VSMC cells (C vs Calcification).

## 4. Discussion

In our present investigation we studied the expression of OC, OPN and RUNX2 in patients’ leucocytes and their possible role as diagnostic markers for cardiovascular disease. Furthermore, calcified human umbilical vein endothelial cells and vascular smooth muscle cells, representing two main components of human vessels, were subjected to transcript and protein analyses with primers and antibodies specific for OC, OPN and RUNX2, respectively. Additionally, the interaction between monocytes and calcified HUVEC and VSMC was investigated in adhesion assays.

The expression of leucocytic OC, OPN and RUNX2 was significantly related to the presence of arteriosclerotic disease, as demonstrated by significantly elevated expression of OPN and RUNX2. The same expression pattern could be induced in calcified HUVEC and VSMC cells. VSMC cells responded to calcification medium with a significantly increased expression of OC, OPN and RUNX2, while in calcified HUVEC OC transcripts were not changed. Immunocytochemistry of calcified HUVEC and VSMC revealed stronger protein expression of OC, OPN and RUNX2 in the calcified cells. The alkaline phosphatase activity was not altered in HUVEC, but significantly increased in VSMC cells treated with calcification medium. At last, the higher number of adhered monocytes was visible in calcified cells as compared to controls. With all these findings, we may conclude that OC, OPN and RUNX2 proved to have a potential diagnostic value in arteriosclerotic disease.

### 4.1. Osteocalcin

Recently, osteocalcin has been described as a potential preventive or therapeutic agent in metabolic disorders [18]. The complex expression of osteocalcin suggests that all its functions are not already known [19]. A recent meta-analysis showed an overall significant inverse association between serum osteocalcin and body mass index [20]. In mouse models and also patients with peripheral artery disease, circulating OC positive mononuclear cells were associated with severe calcification of the aorta [21]. Circulating OC was also associated with plaque destabilization in patients with early coronary atherosclerosis [22]. In a recent study from Ramirez-Sandoval et al., in patients undergoing peritoneal dialysis, there was no significant association between vascular calcification and OC [23]. In our study, OC expression was significantly reduced in patients with arteriosclerotic disease. Our findings are not consistent with the current evidence, and further investigation is needed in order to access these differences.

### 4.2. Osteopontin

In our study, the leucocytic OPN transcripts were significantly elevated in PAD+ patients. OPN is considered a potential therapeutic target in these patients [24]. Lok et al. showed that OPN in lower concentrations may have a protecting effect and in high levels a damaging role in vascular tissue injury [25]. Also, elevated plasma levels of OPN have been discussed as an independent predictor of coronary calcification in patients with diabetes and asymptomatic coronary disease [26]. Further studies investigating OPN mechanisms and relations with the vascular tissue are required to understand if this protein represents a protective or a harmful role in PAD+ patients.

### 4.3. Runt-Related Transcription Factor 2

Runx2 expression is increased in calcifying human atherosclerotic plaques [16]. Additionally, in another study, Tanaka et al. stated that RUNX2 has a major role in osteogenic conversion in atherosclerotic lesions [27]. In mice models, RUNX2 was strongly correlated with the vessel calcification process [28,29]. These findings are consistent with our analysis, which revealed that RUNX2 transcripts were significantly elevated in PAD+ patients.

### 4.4. Alkaline Phosphatase Activity (ALP)

Concerning ALP, our results are consistent with the current literature. In our study, the alkaline phosphatase activity was increased in VSMC cells treated with calcification medium.

In animal models, an increased concentration of ALP in vascular smooth muscle cells or in endothelial cells leads to calcification [30,31]. In patients with coronary artery disease, elevated ALP activity was independently associated with the risk of 3-year all-cause mortality [16]. In a separate study, the overexpression of ALP in vascular endothelium in mice resulted in a different course of atherosclerosis. Calcification preceded lipid deposition [32].

### 4.5. Monocytes

Several studies are trying to identify the monocyte adhesion and plaque recruitment as a potential target for treating arteriosclerosis [33,34]. Here, we confirm this potential as a higher number of adhered monocytes was visible in calcified cells when compared to controls.

### 4.6. Limitations

By design, the assessment of the leucocytic parameters was possible on the transcript level only. In addition, whether the circulating blood levels of OC, OPN and RUNX-2 may support the development of a serum/plasma noninvasive assay useful for diagnostics remains an open question. Furthermore, the patient collective in our study was relatively small. The lack of functional leucocytic data, which is a noticeable limitation of this study, should be addressed in future clinical trials with larger patient cohorts as the biological rationale of OC, OPN and RUNX-2 remains not entirely understood for atherosclerotic disease. All in all, further investigations should be conducted to understand the role of monocytes and alkaline phosphatase activity on the calcification process.

## 5. Conclusions

In this study, a clear relation between symptomatic arteriosclerotic disease and the expression of OC, OPN and RUNX2 in patients’ leucocytes could be demonstrated. It is still not clear if one could be used as a diagnostic marker or a potential drug target. Further studies with larger patient collectives should be designed to address these questions.

## Figures and Tables

**Table 1:** Primer Pairs for the amplifications of target gene transcripts.

Primer	Sequence	Product Size
RUNX2	Sense-5′-CCCTGAACTCTGCACCAAGT-3′ Antisense-5′-GGCTCAGGTAGGAGGGGTAA-3′	120 bp
Osteopontin	s GCCGAGGTGATAGTGTGGTT as AACGGGGATGGCCTTGTATG	149 bp
ßActin	s agg cac cag ggc gtg at as gcc cac ata gga atc ctt ctg ac	51 bp
GAPDH	s acc cag aag act gtg gat gg as ttc tag acg gca ggt cag gt	233 bp
18S	s gtt ggt gga gcg att tgt ctg g as agg gca ggg act taa tca acg c	151 bp

## Data Availability

Not applicable.

## Supplementary Materials

The following are available online at https://www.mdpi.com/2079-9721/9/1/19/s1.

## Sample Availability:

Samples of the compounds are not available from the authors.
